# Meta-analysis of muscle transcriptome data identifies key genes influencing intramuscular fat content in pigs

**DOI:** 10.5713/ab.24.0905

**Published:** 2025-04-28

**Authors:** Yan Wang, Jiahao Wang, Yanhui Zhao, Xihui Sheng, Xiaolong Qi, Lei Zhou, Jianfeng Liu, Chuduan Wang, Jianliang Wu, Yongchun Cao, Kai Xing

**Affiliations:** 1 State Key Laboratory of animal Biotech Breeding, Key Laboratory of Animal Genetics, Breeding and Reproduction, Ministry of Agriculture, College of Animal Science and Technology, China Agricultural University, Beijing, China; 2 College of Animal Science and Technology, Beijing University of Agricultural, Beijing, China; 3 Beijing Zhongyu Pig Breeding Co., Ltd., Beijing, China

**Keywords:** Intramuscular Fat, Meta-analysis, Pig, RNA-Seq

## Abstract

**Objective:**

The content of intramuscular fat (IMF) is closely linked to meat quality, and the mechanism of IMF deposition is complex. Despite numerous transcriptomic studies on IMF, variations in sample sizes and data analysis methods have produced inconsistent gene expression patterns and results. To identify the pivotal genes influencing pig IMF content, we performed a meta-analysis on 10 pig muscle transcriptome datasets with a total of eighty samples, forty with high and forty with low IMF samples.

**Methods:**

DESeq2 has been used to analyze the high and low IMF groups for 10 datasets each, resulting in the differentially expressed genes (DEGs) for each dataset. To identify key genes affecting IMF content, we performed a meta-analysis of the differential expression results from the 10 datasets using MetaVolcanoR. Subsequently, we conducted protein-protein interaction network analysis, Gene Ontology and Kyoto encyclopedia of genes and genomes functional enrichment analysis, and quantitative trait locus (QTL) analysis on the DEGs.

**Results:**

The meta-analysis identified 129 DEGs, comprising 71 upregulated and 58 downregulated DEGs in the high IMF group. The DEGs exhibited enrichment in processes associated with adipocyte differentiation and fat anabolism. QTL analysis demonstrated that five DEGs, including *FASN* and *SCD*, corresponded to six QTLs associated with IMF.

**Conclusion:**

The findings suggest that meta-analysis effectively integrates data from multiple datasets, resulting in more reliable outcomes. This approach enabled the identification of the core gene cluster comprising *FASN*, *SCD*, and *PLIN1*, *LEP*, and *G0S2*, which influence IMF content in pigs.

## INTRODUCTION

Intramuscular fat (IMF) is a vital economic trait in pigs, strongly linked to meat quality, including tenderness, water retention, and flavor [1–3]. The breeding of modern pig breeds for faster growth, higher lean meat percentages, and reduced backfat thickness often reduces IMF content, compromising meat quality. With the improvement of living standards, the growing demand for nutritious and delicious pork has made improving pork quality a key goal in pig genetic breeding.

Current research has identified numerous genes and pathways involved in the complex process of IMF deposition, offering insights into improving meat quality of pig. Sequencing of the *longissimus dorsi* muscle transcriptome of Wannanhua and Yorkshire pigs revealed that the DEGs of the two pig breeds may play an important role in meat quality traits [4]. In a study by Miao et al, 1632 DEGs were identified in the intramuscular adipose tissue of Jinhua and Landrace pigs, indicating significant differences in fat and protein metabolism at the transcriptome level between these two breeds [5]. Another study by Li et al, using transcriptome analysis, suggested that *PLIN1* is a key gene affecting the IMF content in pigs [6]. Xu et al identified 717 genes affecting IMF content in the *longissimus dorsi* muscle of Wei and Yorkshire pigs, mainly involved in pathways like fatty acid metabolism and steroid biosynthesis that regulate fat deposition [7]. Zhang et al identified 85 genes affecting IMF content in Dingyuan pigs, primarily involved in the insulin pathway [8]. Wang et al identified 15 candidate genes affecting fat deposition in Nanyang black pigs, including *FASN*, *CAT*, and *SLC25A20* [9]. However, it should be noted that there is either no overlap or only partial overlap between the DEGs reported in different studies. This discrepancy can be attributed to various factors, including differences in species or tissues, the number of biological repeats, sampling methods, and the use of different bioinformatics pipelines. It is worth mentioning that even small changes in gene expression can have a significant impact on the final results when experimental responses are consistent [10]. Consequently, the reliability of each individual study’s findings remains uncertain, and it is possible that some DEGs with minor responses may have been overlooked. To address these limitations, meta-analysis has become a widely adopted approach, as it can overcome the aforementioned shortcomings and provide more robust and reliable results.

Landrace pigs are famous for their high lean meat percentage and are widely used in commercial pork production, but their IMF content is typically below 2% [11]. Songliao Black Pig is a Chinese lean-type breed with approximately 46% Duroc, 27% Landrace, and 27% Northeast Min Pig bloodlines, also has an IMF content exceeding 3.5% [12,13]. Thus, these two pig breeds provide an excellent comparison for studying growth performance and meat quality in pigs. In this study, we integrated our transcriptome datasets from the *longissimus dorsi* muscle of six Songliao Black pigs and six Landrace pigs with nine IMF-related transcriptome datasets from National Center for Biotechnology Information (NCBI) for a meta-analysis to identify DEGs associated with varying IMF contents in pigs. Functional enrichment and quantitative trait locus (QTL) analyses were conducted to further explore the roles of these DEGs. The objective is to identify key genes influencing IMF content in pigs, uncover the molecular basis of fat deposition, and provide a reference for future breeding efforts.

## MATERIALS AND METHODS

### Ethical statements

The study was approved by the Animal Welfare Committee of China Agricultural University (Permit Number: DK996). During these experiments, every effort was made to minimize pain and discomfort to the animals. The study was conducted in accordance with the ARRIVE guidelines for reporting animal experiments.

### Transcriptome data of this study

We collected 53 Songliao Black pigs and 132 Landrace pigs from the Ninghe Breeding Farm in Tianjin to evaluate their IMF content. All pigs were kept healthy with free access to food and water, raised under identical conditions, and had their IMF content measured upon reaching approximately 100 kg. The IMF content of Songliao Black pigs (2.8±1.28) was significantly higher (p<0.01) than that of Landrace pigs (1.28±0.36). We selected six Songliao Black pigs and six Landrace pigs from each breed for subsequent research. *Longissimus dorsi* muscle tissue was collected from the region between the third-to-last and fourth-to-last ribs and preserved in liquid nitrogen for subsequent RNA extraction.

Total RNA was extracted using the Trizol method, and the quality and integrity of the RNA were verified using 1% agarose gel electrophoresis. Additionally, the concentration and quality of the RNA were further confirmed by measuring the absorbance ratios at 260nm and 280nm using a Smart Spec Plus spectrophotometer (Bio-rad, Hercules, CA, USA). The RNA integrity number was assessed using the Agilent 2100 Bioanalyzer, which provides a quantitative measure of RNA degradation and overall quality. Following the instructions provided by the reverse transcription kit (Invitrogen, Carlsbad, CA, USA), cDNA libraries were prepared from the extracted RNA. After enrichment and purification, sequencing was performed using the Illumina HiSeq 2000 platform (Illumina, San Diego, CA, USA).

### Public transcriptome data

To acquire relevant data pertaining to pig IMF, a search was conducted on NCBI Sequence Read Archive and PubMed using the keywords “pig”, “IMF” and “RNA-seq”. The following inclusion criteria considered while selecting the dataset:(1) The selected research is pig muscle tissue; (2) The data is derived from the transcriptome sequencing platform; (3) Each dataset comprises more than three samples;(4) Each study includes a grouping of high and low IMF; (5) Individual sample expression should be available for download.

### Transcriptome data analysis

Raw data were filtered using fastp (v0.12.4) [14], and the quality of sequencing data was evaluated using FastQC (v0.11.9) [15]. The pig reference genome sequence (version: Sus scrofa v. 11.1) and the genome annotation file were downloaded from the Ensembl database (https://asia.ensembl.org/Sus_scrofa/Info/Index). The index was constructed using Hisat 2 (v2.2.1) [16], and the clean reads were aligned with the reference genome, mRNA quantification was performed using HTseq (v2.0.1) [17] software, and the R software package DESeq2 [18] was employed for gene differential expression analysis. Genes that satisfy p<0.05 and |log_2_(Fold Change)|≥1 are defined as differentially expressed genes (DEGs).

### Meta-analysis

The random effect model (REM) in the R software package MetavolcanoR was used to perform meta-analysis on the differential expression results of each dataset. Following the input requirements of MetavolcanoR, we combined the DESeq2 output from each dataset into a list to serve as the input data for MetavolcanoR, enabling the identification of genes with consistent changes. The REM integrates fold-change and their corresponding variances from each study, applying a weighted method to calculate a summary fold-change. This approach ensures that each study’s contribution is accurately reflected while accounting for study-specific variability. Additionally, REM evaluates heterogeneity among studies using Cochran’s Q statistics, providing insights into the consistency or potential biases across datasets. Finally, the REM estimates a summary p-value, representing the probability that the summary fold-change is not different from zero.

### Function enrichment analysis

To gain a deeper understanding of the functions of DEGs obtained through meta-analysis, we utilized the clusterProfiler R software package [19] to perform enrichment analysis of Gene Ontology (GO) terms and Kyoto Encyclopedia of Genes and Genomes (KEGG) pathways, employing the hypergeometric test for both analyses. Furthermore, we utilized the STRING database [20] to explore protein interactions among the DEGs.

### Quantitative trait locus analysis of differentially expressed genes

To identify candidate genes associated with pig IMF trait, we performed QTL analysis on DEGs. The chromosome location information of the DEGs was obtained from the pig genome annotation file. Additionally, the QTL information related to pig IMF was downloaded from the Animal QTL database [21] (http://www.animalgenome.org/QTLdb). After that, the DEGs were intersected with the chromosome position information of the downloaded QTLs taken using the bedtools [22] function in Linux.

## RESULTS

### Overview of transcriptome datasets

In our self-tested data, the mean IMF content was 3.60 (standard deviation [SD] = 1.23%) for Songliao black pigs and 1.36% (SD = 0.16%) for Landrace pigs. We collected 9 transcriptome datasets of pig muscle with high IMF and low IMF contents that met our criteria, along with our own data, resulting in a total of 80 muscle tissue samples for subsequent analysis (Supplements 1, 2). Although these studies categorize the samples into high IMF and low IMF groups, they include data from different pig breeds, and the slaughter age and gender are also inconsistent.

**Figure 1:**
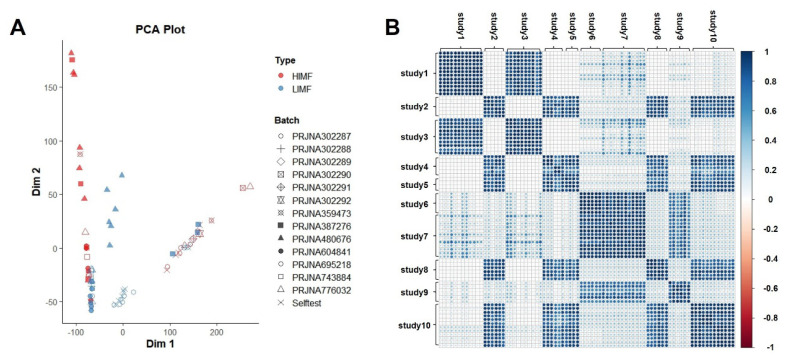
Relationship between samples. A. Principal component analysis (PCA) Visualization of Gene Expression Level Matrices Across Diverse Datasets. B. Correlation diagram between dataset samples. HIMF, high IMF; LIMF, low IMF; IMF, intramuscular fat.

The total number of raw reads, the number of clean reads, and the ratio of transcriptome data to reference genome-mapped reads for the ten studies are shown below (Supplement 3). Principal component analysis (PCA) of the expression levels across different datasets revealed that the samples clustered primarily according to their study origin, indicating substantial heterogeneity between datasets from different studies (Figure 1A). Furthermore, we performed correlation analysis across all samples and found strong correlations within each dataset, but most samples between different datasets showed weak correlations (Figure 1B).

### Differential expression analysis for each dataset

**Figure 2:**
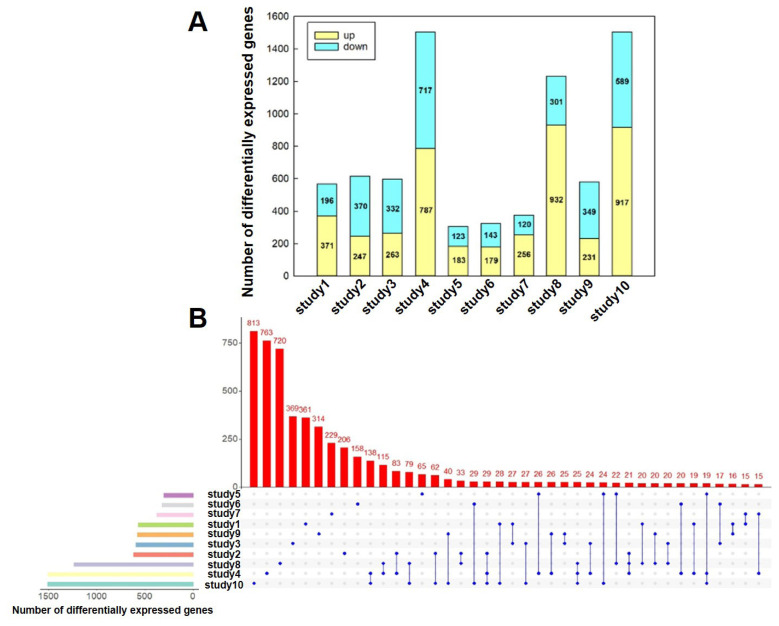
Number and coincidence of DEGs in different data sets. A. Number of DEGs of each dataset. B. Upset diagram of each dataset DEGs. DEGs, differentially expressed genes.

Each dataset divided the samples into high IMF and low IMF groups. We utilized DESeq2 to identify the DEGs for each dataset, resulting in a considerable variation in the number of DEGs across datasets (Figure 2A; Supplement 4). Datasets with many DEGs, such as study10 (our self-tested) and study4 (PRJNA302287) datasets can contain over a thousand genes, while those with fewer DEGs, like study5 (PRJNA359473) and study6 (PRJNA387276) datasets, typically include around 300 genes (Figure 2A; Supplement 4). Further, we compared the common DEGs between datasets. The majority of DEGs were exclusive to their respective studies, with only a few overlapping across two or three datasets and no gene overlap was found in all datasets (Figure 2B).

### Meta-analysis of all datasets

**Figure 3:**
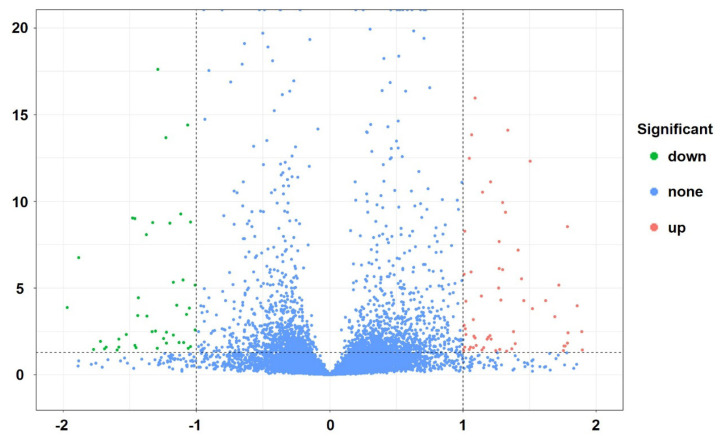
Volcano plot of differentially expressed genes in transcriptome meta-analysis. Red indicates up-regulated expression in the high IMF group, blue indicates down-regulated expression, and gray indicates no significant difference in expression. IMF, intramuscular fat.

Meta-analysis integrates findings from multiple studies by considering the sample size and standard deviation of each, enabling a deeper understanding of research results and generating robust conclusions [23]. In order to integrate the information of the 10 datasets to identify the important genes affecting the amount of IMF, we used the REM method of MetavolcanoR to estimate the summary p-value and summary fold-change. Out of a total of 16,200 tested genes, we identified 129 DEGs (p<0.05 and |log_2_(Fold Change)|≥1). Among these DEGs, 71 were up-regulated and 58 were down-regulated in the high IMF group (Figure 3; Supplement 5).

### Function enrichment and protein-protein interaction network analysis

**Figure 4:**
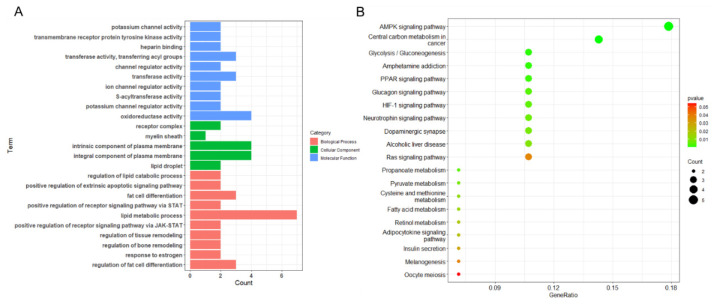
Functional enrichment analysis of meta-analyzed DGEs. A. GO enrichment analysis of DEGs. B. KEGG pathway enrichment analysis of DEGs. DEGs, differentially expressed genes; GO, Gene Ontology; KEGG, Kyoto encyclopedia of genes and genomes.

To better understand the effects of the 129 DEGs on IMF content, we performed functional enrichment analysis of the genes using the R package clusterProfiler. The results of the GO analysis reveal that the biological processes associated with the DEGs are primarily enriched in lipid metabolism, and fat cell differentiation, as well as their regulatory processes (Figure 4A). Regarding molecular functions, the DEGs are primarily linked to membrane protein activity, ion channel activity, and transferase activity (Figure 4A). Furthermore, the cellular components associated with these DEGs are related to the composition of lipid droplets (LDs) and plasma membranes (Figure 4A). The KEGG pathway enrichment analysis revealed that the DEGs were significantly enriched in the AMP-activated protein kinase signaling pathway, Fatty acid metabolism, and Adipocytokine signaling pathway (Figure 4B). These results indicate that the DGEs identified in our meta-analysis are closely associated with IMF content in pigs.

**Figure 5:**
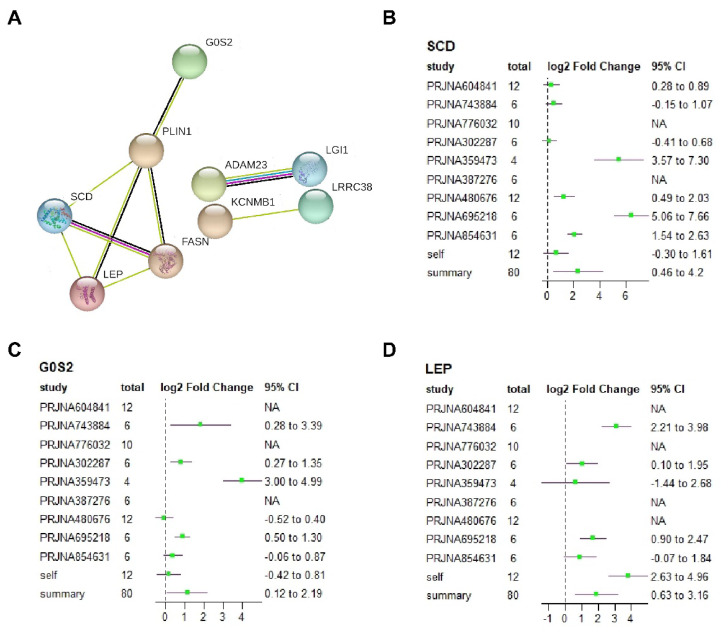
Protein-Protein Interaction Networks analysis to identification of gene clusters. A. Protein-Protein Interaction Networks of DEGs. B–D. Status of differentially expressed genes (DEGs) identified through meta-analysis across different datasets.

To identify the core gene clusters influencing IMF content, we conducted a Protein-Protein Interaction Networks (PPI) analysis using the STRING database [20]. Most proteins did not interact with each other, but we identified a cluster consisting of *SCD*, *LEP*, *FASN*, *PLIN1*, and *G0S2* (Figure 5A). Furthermore, the gene forest plot revealed that, despite some heterogeneity in expression trends across different studies, these genes related to fat deposition were consistently upregulated (Figures 5B–5D; Supplement 6).

### Joint analysis of quantitative trait loci and differentially expressed genes location

To identify potential genes associated with pig IMF traits, we conducted QTL analysis on DEGs. The results of the QTL analysis reveal that 19 IMF content QTLs correspond to 12 DEGs, which include notable genes *FASN* and *SCD* (Table 1).

## DISCUSSION

As consumers increasingly demand healthier pork with preserved organoleptic qualities, IMF content has become a key focus in pig genetic breeding. Although there are many studies on IMF content in pork, the key genes identified in different studies are rarely consistent. Therefore, we collected 10 pig muscle transcriptome datasets for meta-analysis to identify key genes influencing IMF content in pigs. We identified a potential core gene cluster affecting IMF content, with the genes *SCD* and *FASN* located within the QTLs associated with IMF.

We obtained a total of 10 DEGs datas from each transcriptome dataset. The number of DEGs identified across different datasets ranged from several hundred to over a thousand. More than one thousand DGEs were identified in the datasets of study 10 (our own test) and study 4 (PRJNA302287), both of which involved comparisons between pig breeds with significant differences in IMF content. Our data set includes Landrace pigs and Songliao Black pigs, both of which are lean-type breeds. However, Songliao Black Pig is a crossbreed of Duroc, Landrace, and Northeast Min Pig, and its IMF content is significantly higher than that of Landrace pigs [11–13]. The study 4 dataset involves a comparison between Wannanhua pigs and Yorkshire pigs. Yorkshire pigs are a lean-type breed, whereas Wannanhua pigs, a Chinese local dual-purpose breed for both meat and fat, have significantly higher IMF content than Yorkshire pigs [24]. The largest number of DEGs was identified in these two studies, likely due to the large genetic differences between the breeds. However, it is important to note that although significant differences in IMF content between breeds reflect breed specificity, there are many other breed-specific traits. Therefore, the DGEs identified between breeds with significant differences in IMF content within a single study may not necessarily be related to IMF content.

There was limited overlap among these DEG datasets, with most genes being unique to individual studies and no DEG shared across four or more datasets. The PCA and correlation analyses also revealed substantial heterogeneity among samples from different datasets. This phenomenon can be attributed to a multifaceted array of factors. Firstly, the experimental conditions and designs vary significantly across different datasets, leading to distinct transcriptional profiles. Secondly, even within the same species, there exists natural variation in gene expression among individuals, which may be influenced by factors such as genetic background, age, sex, health status, and other biological variables. These factors are important reasons for the identification of varying numbers of DGEs and for limiting the identification of common IMF-affecting genes across different studies.

Meta-analysis aims to analyze and integrate findings from multiple studies to achieve a deeper understanding of research results. It generates robust conclusions by considering the sample size and standard deviation of each study [23]. The REM of MetavolcanoR integrates fold-change and their corresponding variances from each study, applying a weighted method to calculate a summary fold-change. This approach identifies genes with consistent trends across studies and can pinpoint genes that are stably associated with IMF content. Therefore, we performed a meta-analysis of 10 transcriptomic datasets (encompassing 16,200 genes) using this method and identified 129 DEGs, with 71 genes upregulated and 58 genes downregulated in pigs with higher IMF levels. The results of GO enrichment analysis and KEGG pathway analysis indicated that these DEGs predominantly participate in biological processes and pathways related to fat synthesis and metabolism. This result shows that the DEGs identified through meta-analysis are closely related to IMF content, confirming the reliability of the meta-analysis findings.

PPI network analysis of DEGs revealed a core cluster of five interacting proteins, with *FASN* and *SCD* genes located within the QTLs of IMF, highlighting the core cluster association with fat deposition. The *FASN* gene encodes fatty acid synthase, a pivotal enzyme involved in the de novo synthesis of fatty acids. Acetyl coenzyme A in the cytoplasm is carboxylated, and then it is catalyzed by *FASN* together with malonyl coenzyme A to form saturated palmitic acid fatty acid [25]. The research indicates that polymorphisms in the *FASN* gene have a significant impact on fatty acid deposition in pigs [26]. Additionally, it has been observed that the expression level of *FASN* in adipose tissue is significantly higher in obese pigs compared to lean pigs [27]. Moreover, the expression level of *FASN* is significantly higher in pigs with high IMF compared to those with low IMF [28], which aligns with our research findings. The *SCD* gene encodes stearyl coenzyme A desaturase, a fatty acid desaturase located in the endoplasmic reticulum membrane [29]. Stearyl coenzyme A desaturase serves as a rate-limiting enzyme that catalyzes the conversion of saturated fatty acids (SFAs) into monounsaturated fatty acids (MUFAs). It introduces carbon-carbon double bonds at the 9th and 10th carbon atoms of SFAs, palmitic acid and stearic acid, to form MUFAs. MUFAs are essential for the biosynthesis of polyunsaturated fatty acids, cholesterol, triglycerides (TGs), and phospholipids [29,30]. Currently, five subtypes of *SCD* (*SCD1*-*5*) have been identified, with *SCD1* and *SCD5* found in pigs [31]. *SCD1* is significantly associated with fat deposition and composition in skeletal muscle [32] and plays a crucial role in the differentiation of pig adipocytes, being significantly induced during adipocyte differentiation [33]. Knockout mice lacking the *SCD1* gene exhibit resistance to diet-induced obesity, accompanied by enhanced metabolic rate and insulin sensitivity [34]. In our study, we found that the *SCD* gene was up-regulated in pigs with high IMF and was associated with two IMF-related QTLs.

Although the other three genes in the core cluster are not in the known IMF QTLs, they are all closely related to fat-related pathways. The *PLIN1* gene encodes the perilipin, which is primarily expressed in adipocytes and plays a critical role in the regulation of lipolysis [35]. It restricts hormone-sensitive lipase, adipose triglyceride lipase (ATGL), and their co-activator CGI-58 from entering LDs to prevent excessive lipolysis [36,37]. Moreover, the formation of LDs mediated by *PLIN1* can promote the activation of sterol regulatory element binding protein-1 (SREBP-1) during adipogenesis, leading to the accumulation of TGs [38]. Knockdown of the *PLIN1* gene reduces the level of TGs and the size of LDs in pig adipocytes [6]. Leptin (LEP), the product of the obesity gene, plays a vital role in food intake regulation and fat decomposition [39,40]. *LEP* gene expression is higher in adipose tissue of obese individuals [41], which is consistent with our study. The *G0S2* gene is involved in regulating the cell cycle and cell proliferation. Studies have shown that *G0S2* is a target gene of PPARγ [42]. It is a highly expressed member of the PPAR family and serves as a key regulator of adipogenesis [43,44]. *G0S2* selectively inhibits the hydrolysis of TGs by ATGL, resulting in the blockage of lipolysis and the accumulation of TGs [45–47]. Therefore, these five genes are considered potential core gene cluster that may influence the IMF content in pigs.

Our objective was to integrate multiple studies, expand the sample size, and thereby ensure the accuracy of our analysis, providing a valuable reference for future researchers. In comparison with previous studies, we have confirmed the association of genes such as *FASN* and *SCD* with IMF, corroborating the reliability of our research. Notably, we have also uncovered *PLIN1*, *LEP* and GOS2 are also closely related to IMF, and together with *FASN* and *SCD*, they form a core gene cluster that regulates IMF. These findings not only enrich the understanding of the transcriptional landscape of IMF but also offer new perspectives and avenues for future research. However, it’s essential to acknowledge that meta-analyses often prioritize published studies over unpublished ones, which can impact the objectivity and accuracy of the outcomes. Currently, there is a paucity of studies on the transcriptional profiling of high and low IMF in pigs. Consequently, in this meta-analysis, we included studies with relatively small datasets. In the future, we will not only need to further validate the identified core gene clusters, but also expand the sample size to better elucidate the genetic factors influencing IMF and provide a genetic foundation for improving IMF in pigs.

## CONCLUSION

In this study, we performed the most comprehensive meta-analysis of porcine muscle transcriptome data on IMF and identified a core gene cluster affecting IMF. Among these, the genes *SCD* and *FASN* are located within the QTLs associated with IMF, while the other three genes, *PLIN1*, *LEP*, and *G0S2*, are not; however, all of these genes are closely linked to IMF deposition. Our findings suggest that the application of meta-analysis proves to be a powerful approach to integrate transcriptomic data and identify key genes associated with complex traits such as IMF content in pigs.

## Figures and Tables

**Table 1:** Differentially expressed genes and their corresponding IMF-related QTLs

Chromosome	Gene_ID	Gene_start	Gene_end	QTL_ID	QTL_start	QTL_end	PUBMED_ID
Chr.1	*NTRK3*	191535300	191925353	147239	191753507	191753511	28728355
Chr.2	*GPR150*	102222131	102223426	12105	73955159	151935871	17697135
Chr.4	*SBSPON*	62756342	62791613	313	4097207	69552602	10430592
Chr.4				3871	55861932	130011401	11808631
Chr.4				309	62257573	71950968	10920235
Chr.4	*SDR16C5*	75586235	75598989	3871	55861932	130011401	11808631
Chr.4				2982	66054985	79245996	16441291
Chr.6	*MT4*	18610593	18613967	367	8115061	35450005	10859367
Chr.6	*RNF207*	67105763	67116510	150	55524105	132574478	12270105
Chr.6				151	55524105	132574478	12270105
Chr.6				4169	61930732	126424116	10754115
Chr.6	*LRRC38*	72935493	72961920	150	55524105	132574478	12270105
Chr.6				151	55524105	132574478	12270105
Chr.6				4169	61930732	126424116	10754115
Chr.6				7231	71156276	77477481	10754115
Chr.6				163	71156276	89863628	11309662
Chr.6				692	71156276	77477481	12693684
Chr.6	*SGIP1*	145822267	146051955	4165	111133631	152819421	10764061
Chr.12	*FASN*	920507	937559	172584	924490	924494	31132591
Chr.12	*MYH13*	55046895	55103427	37681	55093695	55093699	22532790
Chr.14	*SCD*	111461560	111478031	213565	111461749	111461753	31652864
Chr.14		111461560	111478031	193140	111474341	111474345	31940936
Chr.17	*SFRP1*	10436426	10489748	147549	10459637	10459641	28728355

## SUPPLEMENTARY MATERIAL

**Supplement 1.** Characteristics of the transcriptome datasets used in this study.

**Supplement 2.** Detailed information on each transcriptome data for meta-analysis.

**Supplement 3.** Summary of raw reads, clean reads, and ratios of transcriptome data to reference genome mapped reads for all samples.

**Supplement 4.** Differential expression analyses results of all datasets.

**Supplement 5.** Differential expression genes analyzed by meta-analysis of transcriptome data.

**Supplement 6.** Status of differentially expressed genes (DEGs) identified through meta-analysis across different datasets.
