# Supplementary material for: Meta-analysis of muscle transcriptome data identifies key genes influencing intramuscular fat content in pigs
Source: Anim Biosci. 2025 Apr 28;38(8):1622–32. doi: 10.5713/ab.24.0905 (PMC12229906; doi:10.5713/ab.24.0905)
Supplement: Supplementary file 6 [file ab-24-0905-Supplementary-6.pdf]

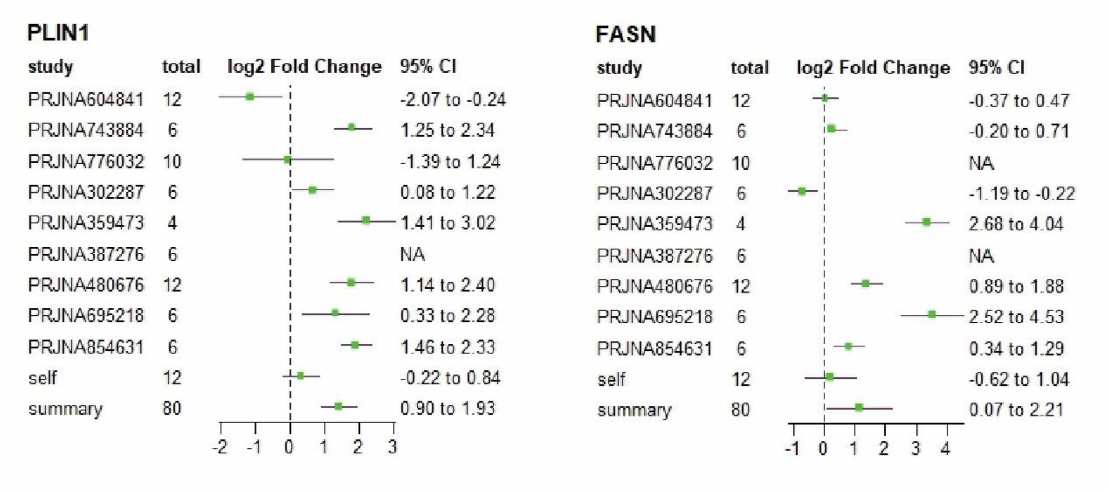

Supplement 6. Status of differentially expressed genes (DEGs) identified through meta-analysis across different datasets.
